# Availability of cancer care services and the organization of care delivery at critical access hospitals

**DOI:** 10.1002/cam4.6337

**Published:** 2023-07-12

**Authors:** Ira S. Moscovice, Helen Parsons, Nathan Bean, Xiomara Santana, Kate Weis, Jane Yuet Ching Hui, Megan Lahr

**Affiliations:** ^1^ Flex Monitoring Team, Division of Health Policy and Management University of Minnesota School of Public Health Minneapolis Minnesota USA; ^2^ Division of Health Policy and Management University of Minnesota School of Public Health Minneapolis Minnesota USA; ^3^ Hennepin County Department of Public Health Minneapolis Minnesota USA; ^4^ University of Minnesota Medical School Minneapolis Minnesota USA; ^5^ Division of Surgical Oncology, Department of Surgery University of Minnesota Minneapolis Minnesota USA

**Keywords:** access, care delivery, critical access hospital, rural, teleoncology

## Abstract

**Introduction:**

Critical access hospitals (CAHs) provide an opportunity to meet the needs of individuals with cancer in rural areas. Two common innovative care delivery methods include the use of traveling oncologists and teleoncology. It is important to understand the availability and organization of cancer care services in CAHs due to the growing population with cancer and expected declines in oncology workforce in rural areas.

**Methods:**

Stratified random sampling was used to generate a sample of 50 CAHs from each of the four U.S. Census Bureau‐designated regions resulting in a total sample of 200 facilities. Analyses were conducted from 135 CAH respondents to understand the availability of cancer care services and organization of cancer care across CAHs.

**Results:**

Almost all CAHs (95%) provided at least one cancer screening or diagnostic service. Forty‐six percent of CAHs reported providing at least one component of cancer treatment (chemotherapy, radiation, or surgery) at their facility. CAHs that offered cancer treatment reported a wide range of health care staff involvement, including 34% of respondents reporting involvement of a local oncologist, 38% reporting involvement of a visiting oncologist, and 28% reporting involvement of a non‐local oncologist using telemedicine.

**Conclusion:**

Growing disparities within rural areas emphasize the importance of ensuring access to timely screening and guideline‐recommended treatment for cancer in rural communities. These data demonstrated that CAHs are addressing the growing need through a variety of approaches including the use of innovative models that utilize non‐local providers and telemedicine to expand access to crucial services for rural residents with cancer.

## 1 INTRODUCTION

Differences in health care access are a significant contributor to the challenges facing cancer patients in rural areas. The distance cancer patients must travel to receive treatment and see health care providers is a major challenge.^1^, ^2^, ^3^ This is largely because only 3% of oncologists in the U.S. work in rural areas.^3^ Longer travel times in rural areas combined with higher rates of financial hardship,^4^, ^5^, ^6^, ^7^, ^8^ lower insurance rates,^4^, ^5^, ^6^, ^9^ lower cancer‐screening rates,^5^, ^7^, ^9^, ^10^ and poor access to standard care^8^, ^11^, ^12^ make it more difficult to access high quality cancer treatment in rural areas.

The cancer care continuum is complex and may include care that extends over an individual's lifetime, from early cancer education and detection through diagnosis, treatment, and survivorship services, all of which may involve a variety of medical personnel and related medical services such as palliative care.^13^, ^14^, ^15^, ^16^ Ideally, patients would have access to necessary cancer care across this continuum at an accessible distance from their home. Critical access hospitals (CAHs) provide one opportunity to meet the needs of individuals with cancer in rural areas. As a Centers for Medicare and Medicaid Services (CMS) designation for eligible rural hospitals with 25 or fewer beds typically located at least 35 miles from another hospital that allows them to receive cost‐based reimbursement, the CAH program aims to improve access to health care and reduce the financial vulnerability of rural hospitals.^17^ For cancer care specifically, prior research has demonstrated that CAHs are less likely to provide chemotherapy, radiation, or comprehensive oncology services than urban hospitals, with one survey from the 2008 to 2017 American Hospital Association demonstrating that only 23% of CAHs offered comprehensive oncology services, 27% offered chemotherapy, and 4% offered radiation, which has declined over time.^18^

While most CAHs do not offer comprehensive oncology services through staff oncologists, they may take advantage of strategies for connecting local patients with non‐local cancer care. Two commonly studied innovative care delivery methods include the use of traveling oncologists and teleoncology, both of which allow rural patients to receive otherwise unavailable specialist care in their local community. Traveling oncologists improve access to cancer care for rural cancer patients by literally delivering care as well as offering education, training, knowledge, and networks to rural providers.^19^, ^20^ Many traveling oncologists provide treatment services that may not be otherwise available in rural health care settings, such as chemotherapy or surgery, utilizing local clinics and hospitals to provide care.^21^, ^22^ Teleoncology, which has increased in use as the COVID‐19 pandemic,^23^, ^24^ can also be an effective tool for reducing the travel burdens that can cause hardship for rural patients and may cause them to forgo treatment entirely.^21^, ^25^ Remote access to oncologists, particularly through video conferencing, can enable access to specialist care during cancer diagnosis and pre‐treatment planning, aid in diagnosis and referrals,^15^, ^26^ and facilitate virtual tumor boards for treatment planning.^27^

With the growing population of individuals residing in rural areas with cancer,^9^ coupled with expected declines in the oncology workforce in rural areas over the coming decades,^28^ it will be important to understand both the availability and the organization of cancer care services in CAHs. The purpose of this study was to provide a current assessment of the availability and organization of cancer care services at CAHs.

## 2 METHODS

### 2.1 Sampling frame

We utilized stratified random sampling to generate a sample of 50 CAHs from each of the four U.S. Census Bureau‐designated regions—the Northeast, the Midwest, the South, and the West—resulting in a total sample of 200 facilities. We then collected contact information for the sampled hospitals and their chief nursing officers (CNOs), including phone numbers and email addresses.

### 2.2 Survey protocol

Using contact information collected online, surveyors called each of the 200 eligible facilities and asked to speak with its CNO. CNOs were allowed to designate a different staff member to respond if they preferred. When a CAH did not have an available CNO, surveyors asked to speak with an employee in a similar position such as a Director of Nursing or Patient Care. If the respondent preferred, due to time availability or other constraints, respondents were offered the option of taking an online version of the survey provided via email. Surveyors initially made up to five completed calls to each hospital. A completed call was any call where the surveyor spoke to the employee of interest or left a voicemail on their direct line. All calls were made at least 2 days apart. After five unsuccessful contacts, non‐responding facilities were switched between surveyors and contacted up to five more times. Near the end of the second round of contacts, researchers used publicly available information to directly email the online survey to non‐respondents, when possible.

### 2.3 Survey Instrument

The survey included 28 items, comprised of multiple choice and open‐ended questions. The survey included queries about the participant and their hospital, the availability of cancer treatment and screenings within their facility, CAH employee involvement in cancer care, hospital policies and practices related to cancer care, and the availability of cancer services such as care coordination, survivorship services, and employee training. Online surveys were completed in Qualtrics, while results from the phone surveys were uploaded into Qualtrics by the surveyors. The initial survey instrument was developed based on guidance from cancer care researchers from the University of Minnesota and feedback from the Federal Office of Rural Health Policy. The survey was pilot tested by two CAH CNOs and revised based on their answers and other feedback before fielding to the 200 eligible CAHs in the study.

### 2.4 Response rate

We received a 68% response rate to the survey fielded between March 1, 2022, and April 30, 2022. Seventy‐nine surveys were completed by phone and 56 were completed using the online survey form. Forty‐seven percent of participants were CNOs, 19% were directors of nursing, and 34% held other positions within their facility (e.g., director of patient care, oncology manager, VP of nursing). Of the remaining CAHs, 63 were not able to be reached, and 2 declined to participate. The response rate was highest among the CAHs in the Northeast region, 78%, while the Midwest, South, and West had response rates of 66, 64, and 62%, respectively.

### 2.5 Analysis

We conducted descriptive statistics to understand variation in availability of cancer care services and organization of cancer care across CAHs responding to the survey. Open‐ended questions were independently coded by two researchers using inductive content analysis to identify important response themes. The coders then compared themes to reach consensus. All analyses were conducted using STATA version 17. The University of Minnesota Institutional Review Board deemed this study not human subjects research. As such, informed consent was not obtained due to the IRB exemption.

## 3 RESULTS

### 3.1 Availability of cancer care services

**FIGURE 1:**
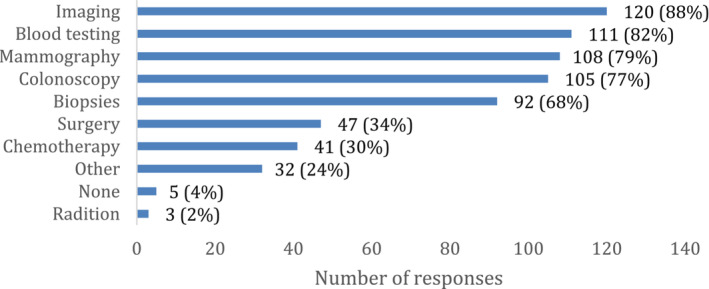
Cancer treatment, diagnostic, and screening services provided at CAHs. Percentages do not sum to 100. Services are not mutually exclusive and CAHs may report more than one type of service.

Nearly half of CAHs (46%) reported providing some component of cancer treatment—chemotherapy, radiation, or surgery—at their facility. CAHs most commonly offered surgery (35%) and chemotherapy (30%), while radiation was much less common (2%) (Figure 1). At CAHs that provided cancer treatment, the most common types of cancer treated were colon cancer (88%) and breast cancer (85%) with lung cancer (44%), prostate cancer (32%), and cervical cancer also being treated.

Fifty‐four percent of CAHs reported not providing any cancer treatments (surgery, radiation, or chemotherapy). Over half of these CAHs (53%) reported the main reason they did not was due to their lack of specialized personnel. Participants reported that when local residents with cancer did not receive treatment at their facility (n = 131) they most often visited the nearest local hospital that offered the necessary care (70%), rather than visiting regional cancer centers (27%) or National Cancer Institute‐designated cancer centers (3%).

It was more common for CAHs to offer cancer screenings and diagnostics than treatment (Figure 1). CAHs commonly offered mammography (80%) and colonoscopy (77%) for cancer screening. Large majorities reported that they provided common diagnostics such as imaging (89%), blood testing (82%), and biopsies (68%). Over half of CAHs provided all of the above listed screening and diagnostics (54%) and only 5% of CAHs did not provide any.

Survey responses were relatively evenly distributed across the four U.S. Census regions. Almost all CAHs (95%) provided at least one cancer screening or diagnostic service and CAHs in the Northeast (58%) and Midwest (52%) were more likely to provide at least one cancer treatment service than CAHs in the West (39%) and the South (31%).

Forty percent of respondents reported that their CAH was owned by a health system. These systems supported CAH cancer treatment in several ways. Among CAHs that were part of a health system and provided cancer treatment, 63% of those health systems provided staff training related to cancer treatment, 60% provided financing for treatment infrastructure, and 48% provided telemedicine services for cancer treatment. Additionally, over 20% of CAHs reported that health care professionals from their hospital participated in tumor boards or cancer care committees.

### 3.2 Organization of cancer care across CAHs

**FIGURE 2:**
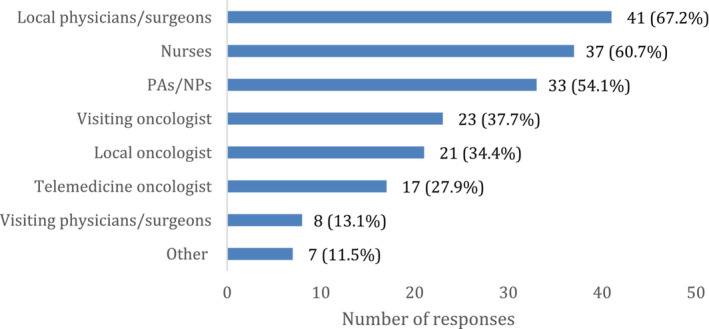
CAH staff involvement in ordering or providing cancer treatment.

CAHs that offered cancer treatment (*n* = 61) reported a wide range of healthcare staff involvement in those services (Figure 2). Specifically, 34% of respondents reported the involvement of a local oncologist, 38% reported the involvement of a visiting oncologist, and 28% reported the involvement of a non‐local oncologist using telemedicine. In addition, most CAHs that provided cancer treatment reported that local physicians and surgeons (67%), nurses (61%), and physician assistants or nurse practitioners (54%) were involved as well.

**FIGURE 3:**
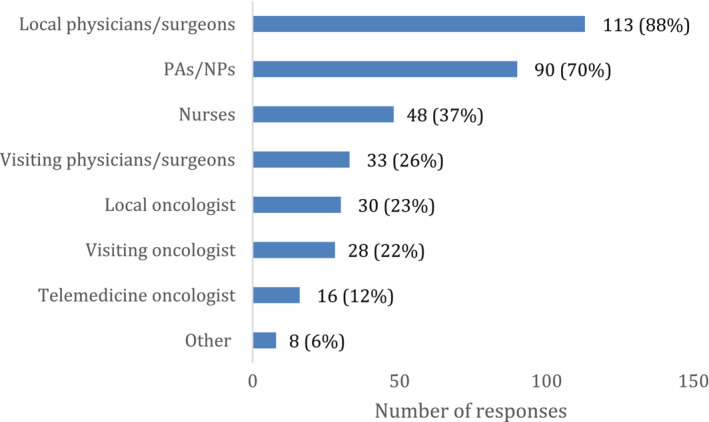
CAH staff involvement in ordering or providing cancer screening or diagnostics.

A smaller portion (44%) of CAHs that offered screening or diagnostics had oncologist involvement in those procedures. Local oncologists were involved in screening and diagnostics in 23% of cases, visiting oncologists in 22% of cases, and non‐local oncologists were involved via telemedicine 12% of the time (Figure 3). Local physicians and surgeons (88%) and physician assistants and nurse practitioners (70%) had more involvement with screenings and diagnostics than with treatment.

Most CAHs (56%) did not report directly employing any healthcare professionals with specialized training or a focus on cancer care. Only 12% of respondents reported directly employing an oncologist at their facility. Facilities were most likely to directly employ nurses (30%) with specialized training than any other types of health professionals.

The use of advanced practice providers (APPs), such as nurse practitioners (NPs) and/or physician assistants (PAs), is an important workforce consideration, particularly in CAHs. Over two‐thirds (67.4%) of CAHs responding to the survey had APPs providing cancer care treatment and/or screening in their facility. Additionally, when looking at CAHs who provided cancer care treatment through the use of visiting oncologists, oncologists via telemedicine, and/or visiting physicians or surgeons (*n* = 34), 70.6% of these CAHs also used APPs to provide cancer care treatment and/or screening.

### 3.3 Level of cancer care services categorization

The information collected on CAH availability of cancer care services and staff involvement in the provision of those services was used to define the following four levels of CAH cancer care services.

1. High (*n* = 26) CAH provides cancer screenings or diagnostics as well as treatment and directly employs an oncologist or has a health professional that participates in a tumor board.
2. Medium (*n* = 36) CAH provides cancer screenings or diagnostics as well as treatment.
3. Low (*n* = 67) CAH provides cancer screenings or diagnostics but not treatment.
4. None (*n* = 7) CAH does not provide cancer screening, diagnostics, or treatment.

Significant variation exists in the level of cancer care services provided at CAHs with slightly less than half of CAHs actively providing a substantial amount (i.e., high or medium level) of cancer care services, half of CAHs providing a low amount of cancer care services which do not involve treatment and very few CAHs that are not providing any cancer care screening, diagnostic or treatment services.

### 3.4 Survivorship services

A minority of respondents (43%) also reported that they offered cancer survivorship services. CAHs most commonly offered late effects screenings (32%), and also offered survivor support groups (15%) and survivor care plans (10%).

## 4 DISCUSSION

While cancer rates have declined nationally, mortality rates from cancer have been higher in rural communities, with rural–urban differences increasing over time.^29^ With this increasing disparity between rural and urban communities, it is critically important to ensure access to guideline‐recommended treatment as well as timely cancer screening in rural areas. This study has shown that CAHs are using innovative models of care to address these challenges such as utilizing non‐local providers and, in a some CAHs, telemedicine, to increase access to these cancer care services for those in rural communities. Additionally, nearly half of CAHs reported providing some component of cancer treatment—chemotherapy, radiation, or surgery—at their facility.

This study suggests cancer screening and treatment services provided by CAHs may not meet the growing demand for these cancer care services in rural communities, with 40% of CAHs in the survey describing work with non‐local providers to provide these services.^30^ While our results highlight a larger proportion of CAHS offering screening services, they also echo previous studies of cancer treatment in CAHs, which indicate that in 2008 less than one‐third of CAHs provided chemotherapy and fewer than 3% provided radiation therapy services.^18^ These results align with the context of the specialized training and equipment required to maintain oncology‐focused care. Whereas the development of models for robust cancer screening services through mobile cancer screening clinics and new, less invasive screening methods (e.g., stool samples for colon cancer) allow for the broader expansion of these services in CAHs,^31^, ^32^, ^33^, ^34^ the relatively fewer number of CAHs offering cancer treatment may occur for a number of reasons. In 2020, roughly two‐thirds of rural counties had no oncologist, which translates to nearly 32 million Americans that do not have access to an oncologist in their county.^28^ With increasing retirements in the near future (21.1% of oncologists are approaching retirement age),^35^ rural communities will need innovative solutions to attend to these shortages.

Cancer treatment in any location is complex, and we recognize that rural residents face additional barriers in adequately gaining access to guideline‐recommended care.^36^, ^37^ Most patients with cancer encounter barriers to care (logistical, financial, and emotional) that significantly affect their ability to receive recommended care. Rural residents are also faced with additional issues, such as childcare issues, transportation challenges, and longer travel distances to care that may exacerbate individual struggles, as well as rural–urban cancer disparities.^28^, ^38^ A number of approaches exist to increase the capacity of hospital staff and providers to address these underlying social determinants of health by partnering with local community organizations that provide support for these services. Our survey identified that CAHs are regularly^39^ collaborating with community organizations to support CAH capacity to meet patients' needs before and during a cancer diagnosis, especially by providing resources for cancer screening, education, and awareness, as well as support services for individuals with cancer and their families. To further enhance this capacity, we can look to previous work on models of cancer care coordination in rural settings, which promote models of cross‐region provider collaboration and care coordination.^40^ In 2019, the American Society of Clinical Oncology hosted a forum titled Closing the Rural Cancer Care Gap, which looked for opportunities and best practices, noting a number of opportunities related to care coordination including working with community leaders to assess local gaps in cancer care and provide community‐based solutions.^21^

Going forward, there are a variety of innovative ways in which CAHs may further confront the cancer care needs of their rural communities, including by building relationships with non‐local providers and using telemedicine to improve barriers in access. The COVID‐19 pandemic drastically changed the delivery of cancer care nationally as oncology providers in urban and rural communities converted to telemedicine.^41^ Despite this change in modality, patients with cancer have also expressed their satisfaction with this changed form of care.^42^ With many telemedicine services continuing to be reimbursed under Medicare and other insurers,^43^ this approach to cancer care may increase access for rural patients and allow for additional capacity and expanded services in CAHs in the future, specifically for consultations.

With the recent end of the COVID‐19 public health emergency earlier this year, policymakers continue to consider a variety of legislation to expand flexibilities in how telehealth services are administered,^44^ including lifting geographic and site‐based restrictions,^45^, ^46^ expanding the scope of providers eligible to provide telehealth services^47^ and implementing permanent coverage of some telehealth expansions.^48^ In order to promote critical access to oncology providers given the predicted reduction in the oncology workforce^35^ and increase in cancer burden within rural areas in the coming decades,^9^ it will be essential for Congress to permanently expand these telehealth flexibilities.

We recognize, however, that telehealth is only one part in helping to meet the needs of rural residents when faced with a cancer diagnosis. In order to meet these needs, CAHs and their providers may consider innovative models of cooperative networks to promote efficiency and access to cancer care services.^49^ Building on the cooperatives concept, rural CAHs could consider similar approaches to shared services and provider retention specifically focused on the growing cancer care needs in rural communities such as provider sharing, rotating clinical services (e.g., mammography), and shared purchasing of oncology‐specific supplies. As access to clinical trials can improve access to state‐of‐the‐art therapy, CAHs may also consider broadening partnerships with one of the National Cancer Institute Community Oncology Research Program (NCORP) sites across the United States, which includes a network of 7 research and 46 community sites (including 14 minority/underserved sites), to bring cancer clinical trials and care delivery to people in their own communities.^50^ Combined, these approaches provide significant potential to create a sustainable model of cancer care access to rural individuals.

While our findings report cancer care delivery practices from a representative sample of CAHs across the United States, we recognize some limitations. First, our results relied on self‐reported data from CAHs about their provision of cancer care services. However, we purposefully surveyed individuals knowledgeable about the care delivery practices across the CAHs, including Chief Nursing and Medical Officers. Additionally, due to the large number of CAHs in the United States, we were not able to survey a complete census of facilities, but we did use a randomized sample stratified by geographic region that received a very high response rate. Finally, we were unable to assess detailed patterns of delivery of specific regimens or specialized treatments (e.g., intensity‐modulated radiation therapy) or staff training (e.g., cancer‐specific continuing medical education for cancer care) due to the broad and diverse range of potential therapies provided to individuals with cancer. We did, however, focus on delivery of the foundational treatments (e.g., surgery, radiation, chemotherapy) to provide key patterns of access across CAHs. These findings provide a starting point for CAHs to engage in conversations for enhanced collaborations to provide ongoing support of rural cancer care.

In conclusion, our assessment of the availability and organization of cancer care services at CAHs found that nearly half of CAHs (46%) reported providing some component of cancer treatment. However, it was more common for CAHs to offer common screenings and diagnostics than treatment. Further, a growing portion of these services were provided by non‐local providers or via telemedicine. These findings can inform providers, policymakers and payers on opportunities to ensure access to high quality cancer care for rural communities.

## AUTHOR CONTRIBUTIONS

**Ira S. Moscovice:** Conceptualization (lead); formal analysis (supporting); funding acquisition (lead); investigation (equal); methodology (equal); supervision (lead); writing – original draft (lead); writing – review and editing (equal). **Helen Parsons:** Conceptualization (equal); methodology (equal); writing – original draft (equal); writing – review and editing (equal). **Nathan Bean:** Conceptualization (equal); data curation (lead); formal analysis (lead); investigation (equal); methodology (equal); project administration (equal); visualization (equal); writing – original draft (supporting); writing – review and editing (supporting). **Xiomara Santana:** Formal analysis (supporting); investigation (equal); methodology (supporting); project administration (supporting); writing – original draft (supporting); writing – review and editing (supporting). **Kate Weis:** Conceptualization (supporting); methodology (supporting); writing – original draft (equal); writing – review and editing (supporting). **Jane Yuet Ching Hui:** Conceptualization (supporting); investigation (supporting); methodology (equal); writing – original draft (supporting); writing – review and editing (supporting). **Megan Lahr:** Conceptualization (equal); data curation (supporting); formal analysis (supporting); funding acquisition (supporting); investigation (supporting); methodology (supporting); project administration (supporting); supervision (supporting); writing – original draft (supporting); writing – review and editing (supporting).

## FUNDING INFORMATION

This study was supported by the Federal Office of Rural Health Policy (FORHP), Health Resources and Services Administration (HRSA), U.S. Department of Health and Human Services (HHS) Cooperative Agreement U27RH01080. The information, conclusions and opinions expressed are those of the authors and no endorsement by FORHP, HRSA, or HHS is intended or should be inferred.

## Data Availability

The authors have decided to not share data.
